# Social and occupational vulnerabilities in people with sickle cell anemia: evidence from a descriptive study

**DOI:** 10.47626/1679-4435-2026-1589

**Published:** 2026-10-20

**Authors:** Mário Cézar de Oliveira, Rosana Paula Pires, João Carlos de Oliveira, Tânia Machado de Alcântara

**Affiliations:** 1 Hospital de Clínicas de Uberlândia, Unidade de Hematologia e Hemoterapia, Uberlândia, MG, Brazil.; 2 Universidade Federal de Uberlândia, Escola Técnica de Saúde, Uberlândia, MG, Brazil.; 3 Hospital de Clínicas de Uberlândia, Laboratório de Patologia, Uberlândia, MG, Brazil.

**Keywords:** sickle cell anemia, social conditions, housing, work

## Abstract

**Introduction:**

Sickle cell anemia is a chronic genetic disorder that affects quality of life and social participation, particularly in the workplace.

**Objectives:**

To describe the living conditions and employment status of working-age adults with sickle cell anemia residing in the municipality of Uberlândia, Minas Gerais, Brazil.

**Methods:**

This descriptive study was conducted between November 2018 and January 2019 among individuals with sickle cell anemia. Socioeconomic, employment-related, and clinical data were collected through semistructured interviews.

**Results:**

A total of 34 participants were included, of whom 70.6% were single, 73.5% lived in households with 1 to 4 residents, 55.9% had access to primary health care, 35.4% lived in rented homes, and 97.1% had access to running water, a sewage system, and regular garbage collection. In addition, 58.8% of participants began working between 12 and 19 years of age, and 52.9% did not have a formal employment contract in their most recent job. Regarding clinical aspects, 67.6% reported up to five crises per year, 82.4% were not receiving psychological follow-up, and 97.1% had been hospitalized at least once. Pain crises were reported by all participants (100%), while 79.4% had experienced pneumonia and 52.9% splenic sequestration.

**Conclusions:**

The findings indicate that sickle cell anemia is associated with substantial social and employment-related limitations. At the time of the study, 67.7% of participants were not working, and 52.9% did not have a formal employment contract in their most recent job, highlighting challenges related to entering and remaining in the labor market.

## Introduction

Sickle cell anemia is a chronic inherited genetic disorder caused by a mutation in the *HBB* gene, leading to the production of hemoglobin S. This alteration causes red blood cells to become deformed under low-oxygen conditions, impairing oxygen transport and resulting in recurrent episodes of pain, infections, chronic anemia, and progressive damage to multiple organs ^[1,2]^. It is the most prevalent genetic disorder in Brazil, particularly among Black and mixed-race populations, reflecting the country’s African ancestry and historically entrenched racial inequalities ^[3,4]^.

In Brazil, it is estimated that thousands of children are born with sickle cell anemia each year, making it a relevant public health concern ^[5]^. Despite advances in early diagnosis through newborn screening and increased access to treatment, morbidity and mortality associated with the disease remain substantial, particularly in regions with limited coverage of specialized health care services ^[3]^.

Social determinants of health, understood as a set of socioeconomic, environmental, and educational factors and access to services, directly influence the course of sickle cell anemia ^[6]^. Low income, limited educational attainment, inadequate housing, and limited access to health care services contribute to worsening clinical complications and poorer quality of life among people with sickle cell anemia ^[6,7]^. Accordingly, individuals with greater social vulnerability tend to experience more frequent pain crises, hospitalizations, and preventable deaths ^[8]^.

In addition, social stigma, structural racism, and the historical invisibility of the Black population within the health care system further intensify the challenges faced by individuals with sickle cell anemia ^[9]^. These barriers affect continuity of care, treatment adherence, and recognition of individual needs, reinforcing the importance of intersectoral approaches in public health policies ^[7]^.

Against this background, this study aimed to describe the living conditions and employment status of working-age individuals with sickle cell anemia residing in the municipality of Uberlândia, Minas Gerais, Brazil.

## Methods

This descriptive cross-sectional study was conducted between November 2018 and January 2019 among individuals with sickle cell anemia aged 18 to 65 years (working age) residing in Uberlândia, Minas Gerais, Brazil. Participants were recruited using the snowball sampling method, as described by Berg ^[10]^.

Descriptive analyses of demographic, socioeconomic, and clinical data were performed based on semistructured interviews conducted after participants had read and signed the informed consent form. The interviews were conducted by a single researcher and lasted approximately 30 minutes.

The sample size was calculated according to Fonseca & Martins ^[11]^. The calculation was based on an estimated population of approximately 400,000 residents of Uberlândia aged 18 to 54 years ^[12]^, a 95% confidence level, a sampling precision of 1%, and an estimated incidence of sickle cell anemia in Minas Gerais of approximately 1 case per 1,400 live births ^[13]^. Based on these assumptions, the minimum required sample size was 27 participants.

The study protocol was approved by the Research Ethics Committee of the Universidade Federal de Uberlândia (UFU) (CAAE 2.985.296).

## Results

The study sample comprised 34 working-age participants (18 to 65 years), of whom 70.6% were single. Most participants lived in households with up to four residents (73.5%) and in homes with three to six rooms (82.3%); 88.2% reported good home ventilation. Regarding household income, households with two income earners were the most frequent (41.2%), whereas households with either one or three income earners each accounted for 29.4%. Only slightly more than half of the participants (55.9%) reported having access to primary health care in their residential area (Table 1).

**Table 1.** Sociodemographic and household characteristics of individuals with sickle cell anemia, November 2018 to January 2019

Characteristic	N (%)
Marital status	
Single	24 (70.6)
Married	10 (29.4)
No. of household residents	
1-4	25 (73.5)
5-7	9 (26.5)
No. of rooms in the home	
3-6	28 (82.3)
7-11	6 (17.7)
Well-ventilated home	
Yes	30 (88.2)
No	4 (11.8)
No. of household income earners	
1	10 (29.4)
2	14 (41.2)
3	10 (29.4)
Access to PHC in the residential area	
Yes	19 (55.9)
No	14 (44.1)

PHC = primary health care.

Regarding housing conditions, rented (35.4%) and owned (32.3%) homes predominated, followed by mortgaged homes (23.5%). With respect to basic household infrastructure, 97.1% of participants reported access to piped water, a sewage system, and regular garbage collection, indicating adequate sanitary conditions (Table 2).

**Table 2.** Housing characteristics of individuals with sickle cell anemia, November 2018 to January 2019

Characteristic	N (%)
Housing tenure	
Rented	12 (35.4)
Assigned	2 (5.9)
Mortgaged	8 (23.5)
Owned	11 (32.3)
Occupancy	1 (2.9)
Piped water	
Yes	33 (97.1)
No	1 (2.9)
Sewage system	
Yes	33 (97.1)
No	1 (2.9)
Garbage collection	
Yes	33 (97.1)
No	1 (2.9)

Regarding employment, more than half of the participants had started working during adolescence, between 12 and 19 years of age (58.8%), whereas 23.5% had never worked. Most participants were not working at the time of the study (67.7%) and were not engaged in paid work (70.6%). Nevertheless, a high proportion expressed interest in learning a new occupational skill (91.2%). Regarding employment history, 29.4% of participants had remained in their previous job for 2 to 5 years, and 14.7% had been in their current job for up to 1 year. In addition, 52.9% reported that they did not have a signed employment record card in their most recent job, indicating a high level of informal employment (Table 3).

**Table 3.** Employment characteristics of individuals with sickle cell anemia, November 2018 to January 2019

Characteristic	N (%)
Age at first job (years)	
Never worked	8 (23.5)
12-19	20 (58.8)
22-28	4 (11.8)
36-38	2 (5.9)
Currently working	
Yes	11 (32.3)
No	23 (67.7)
Interested in learning a new occupational skill	
Yes	31 (91.2)
No	3 (8.8)
Currently engaged in paid work	
Yes	10 (29.4)
No	24 (70.6)
Duration of previous job (years)	
Up to 1 year	5 (14.7)
2-5	10 (29.4)
6-8	5 (14.7)
>15	3 (8.8)
Duration of current job (years)	
Up to 1 year	5 (14.7)
2-4	2 (5.9)
>9	2 (5.9)
Signed employment record card in most recent job	
Yes	15 (44.1)
No	18 (52.9)

Most participants were diagnosed with sickle cell anemia early in life, with 23.5% diagnosed at birth and 44.1% by 10 years of age. Overall, 67.6% reported up to five crises per year, whereas 32.4% reported six or more. Adherence to home treatment was high (88.2%); however, most participants were not receiving psychological follow-up (82.4%). Nearly all participants had been hospitalized at least once (97.1%), and 73.5% reported more than 50 hospitalizations over their lifetime. After the onset of pain, 29.4% of participants reported seeking medical care within a few hours; 88.2% had no other diseases unrelated to sickle cell anemia (Table 4).

**Table 4.** Clinical characteristics of individuals with sickle cell anemia, November 2018 to January 2019

Characteristic	N (%)
Age at diagnosis (years)	
At birth	8 (23.5)
Up to 1	6 (17.6)
2-10	9 (26.5)
11-20	6 (17.6)
≥ 21	5 (14.7)
No. of crises per year	
0-5	23 (67.6)
6-10	7 (20.6)
12-20	4 (11.8)
Home treatment	
Yes	30 (88.2)
No	4 (11.8)
Psychological follow-up	
Yes	
No	6 (17.6)
Has been hospitalized	
Yes	33 (97.1)
No	1 (2.9)
No. of hospitalizations	
< 50	9 (26.5)
> 50	25 (73.5)
Time to seeking medical care after pain onset	
Did not seek medical care	5 (14.7)
Within hours	10 (29.4)
1 day	7 (20.6)
2 days	8 (23.5)
1 week	4 (11.8)
Other disease unrelated to sickle cell anemia	
Yes	4 (11.8)
No	30 (88.2)
Clinical manifestations	
Splenic sequestration	18 (52.9)
Pain crises	34(100.0)
Pneumonia	27 (79.4)
Osteonecrosis	14 (41.2)
Priapism	8 (23.5)
Penile necrosis	2 (5.8)
Acute chest syndrome	11 (32.6)
Cholelithiasis	15 (44.1)
Stroke	8 (23.5)
Aplastic crisis	1 (2.9)
Iron overload	12 (35.3)
Miscarriage	1 (2.9)
Pulmonary hypertension	6 (17.6)
Heart disease	3 (8.8)
Osteomyelitis	1 (2.9)
Chronic kidney disease	3 (8.8)
Leg ulcers	9 (26.5)

Complications related to sickle cell anemia were frequent and varied, with pain crises reported by all participants (100%). Other common complications included pneumonia (79.4%), splenic sequestration (52.9%), cholelithiasis (44.1%), and osteonecrosis (41.2%). Severe complications, such as acute chest syndrome (32.6%), stroke (23.5%), priapism (23.5%), and leg ulcers (26.5%), were also reported, underscoring the substantial impact of the disease on the health and quality of life of the individuals evaluated (Table 4).

## Discussion

The results of this study show that individuals with sickle cell anemia face substantial social, employment, and clinical vulnerabilities. The predominance of single participants and of individuals living in households with up to four residents reflects a relatively small household context, which may correspond to different family arrangements and forms of social support organization. This may negatively affect coping with the disease and continuity of care, as previously reported in the literature on social determinants of health ^[6,14]^.

Although most participants lived in homes with an adequate number of rooms and good ventilation, nearly half reported a lack of primary health care services in their residential area. This finding is concerning, given that primary health care plays a central role in the longitudinal management of sickle cell anemia, prevention of complications, and reduction of avoidable hospitalizations ^[4]^.

Housing conditions were generally satisfactory with respect to sanitary infrastructure, with widespread access to piped water, sewage systems, and garbage collection. Nevertheless, studies suggest that the mere presence of basic infrastructure does not eliminate inequalities in access to specialized health care services, particularly among historically marginalized populations, such as people with sickle cell anemia in Brazil ^[3]^.

Regarding employment, more than half of the participants started working during adolescence, while a substantial proportion had never worked. This early entry into the labor market may be related to economic necessity, whereas the lack of work experience among some individuals may reflect limitations imposed by the disease itself, such as frequent pain crises and recurrent hospitalizations ^[15]^.

The high proportion of participants who were not working at the time of the study and were not engaged in paid work highlights the substantial impact of sickle cell anemia on work capacity. The unpredictability of crises, absenteeism, and physical limitations have been shown to hinder both entering and remaining in the formal labor market ^[16,17]^.

Another relevant finding was the high prevalence of informal employment, with more than half of the participants reporting that their most recent job was not covered by a formal employment contract. Precarious employment increases social vulnerability, limits access to social security benefits, and exacerbates financial insecurity, creating a cycle that may further compromise treatment adherence and self-care ^[7]^. Despite these difficulties, participants showed a high level of interest in learning a new occupational skill, indicating the need for public policies aimed at vocational rehabilitation and labor market inclusion of people with sickle cell anemia. Such policies should take into account disease-related physical limitations and the need for flexible and adapted work environments ^[18]^.

Regarding clinical aspects, most participants were diagnosed during childhood, reflecting advances associated with the National Newborn Screening Program. However, despite early diagnosis, high rates of pain crises and hospitalizations persist, indicating gaps in the effectiveness of continuous follow-up and timely access to specialized care ^[4]^.

The high frequency of hospitalizations, with a large proportion of participants reporting more than 50 episodes over their lifetime, reflects the substantial morbidity associated with sickle cell anemia. This pattern of repeated hospitalizations negatively affects quality of life, autonomy, and social participation, while also placing a burden on the health care system ^[3]^.

Another concerning finding is the low uptake of psychological follow-up, despite the substantial emotional burden and chronic suffering associated with the disease. The absence of psychological support has been associated with poorer pain management, greater emotional distress, and lower treatment adherence, reinforcing the need for comprehensive, multidisciplinary care ^[17]^.

The clinical complications identified, including pneumonia, splenic sequestration, osteonecrosis, acute chest syndrome, and stroke, are consistent with the natural history of sickle cell anemia described in other studies. The high prevalence of these complications may help explain the functional limitations and adverse impacts on employment observed in the study sample, as well as the high need for hospitalizations ^[4]^.

Among the limitations of this study is the difficulty in locating and recruiting individuals with sickle cell anemia. The limited availability of participants who met the inclusion criteria, together with difficulties in accessing and contacting this population, resulted in a small sample size. This limitation may restrict the generalizability of the findings to the broader population of individuals with sickle cell anemia. Nevertheless, the data obtained contribute to expanding knowledge of the characteristics and vulnerabilities of this population and may inform future studies with larger samples and different recruitment strategies.

Finally, the findings of this study show that the impact of sickle cell anemia extends beyond the clinical dimension, encompassing social and employment-related aspects that may influence the living conditions of affected individuals. With regard to employment, 67.7% of participants were not working at the time of the study and only 29.4% were engaged in paid work, although 91.2% expressed interest in learning a new occupational skill. These findings point to challenges related to entering and remaining in the labor market, highlighting the importance of strategies that promote productive inclusion for this population.

Accordingly, sickle cell anemia should be understood as a condition that encompasses clinical, social, and employment-related dimensions, taking into account the multiple vulnerabilities that may shape the experience of living with the disease. The implementation of intersectoral public policies involving health, employment, social assistance, and education is essential to expand access to comprehensive care, promote productive inclusion, and reduce inequalities. In this context, initiatives aimed at vocational training, protection of labor rights, and multidisciplinary follow-up may contribute to promoting equity and improving the living conditions of individuals with sickle cell anemia.

## Data Availability

The data supporting the findings of this study are available within the article.
